# The Exploration of the *Thermococcus barophilus* Lipidome Reveals the Widest Variety of Phosphoglycolipids in Thermococcales

**DOI:** 10.3389/fmicb.2022.869479

**Published:** 2022-07-05

**Authors:** Maxime Tourte, Sarah Coffinet, Lars Wörmer, Julius S. Lipp, Kai-Uwe Hinrichs, Philippe M. Oger

**Affiliations:** ^1^Univ. Lyon, Univ. Lyon 1, CNRS, UMR 5240, Villeurbanne, France; ^2^Univ. Lyon, INSA Lyon, CNRS, UMR 5240, Villeurbanne, France; ^3^MARUM Center for Marine Environmental Sciences, University of Bremen, Bremen, Germany

**Keywords:** archaea, archaeal lipids, intact polar lipids (IPL), adaptation to extreme environments, *Thermococcus barophilus*, diether lipid, tetraether lipid, lipid extraction

## Abstract

One of the most distinctive characteristics of archaea is their unique lipids. While the general nature of archaeal lipids has been linked to their tolerance to extreme conditions, little is known about the diversity of lipidic structures archaea are able to synthesize, which hinders the elucidation of the physicochemical properties of their cell membrane. In an effort to widen the known lipid repertoire of the piezophilic and hyperthermophilic model archaeon *Thermococcus barophilus*, we comprehensively characterized its intact polar lipid (IPL), core lipid (CL), and polar head group compositions using a combination of cutting-edge liquid chromatography and mass spectrometric ionization systems. We tentatively identified 82 different IPLs based on five distinct CLs and 10 polar head group derivatives of phosphatidylhexoses, including compounds reported here for the first time, e.g., di-N-acetylhexosamine phosphatidylhexose-bearing lipids. Despite having extended the knowledge on the lipidome, our results also indicate that the majority of *T. barophilus* lipids remain inaccessible to current analytical procedures and that improvements in lipid extraction and analysis are still required. This expanded yet incomplete lipidome nonetheless opens new avenues for understanding the physiology, physicochemical properties, and organization of the membrane in this archaeon as well as other archaea.

## Introduction

Membranes provide the cells of the three domains of life, eukarya, bacteria, and archaea, with dynamic physical boundaries between the inside and the outside worlds. While their primary function is to ensure cellular integrity, biological membranes are much more than simple barriers. They regulate inward and outward fluxes; support signal transduction, cell bioenergetics, and cell-to-cell communications; control cell shape, growth, and division; and deform to generate, release, and accept vesicles and other membrane macrostructures. These two-dimensional structures are mixtures of a myriad of lipids and proteins that are compositionally, functionally, and structurally complex. In Eukarya, membranes are laterally organized into nano- to microscopic domains with specific compositions, physicochemical properties, and functions formerly termed lipid rafts (Kraft, 2013; Nickels et al., 2019). Such a membrane organization is essential for membrane-hosted cellular functions, as it facilitates the organization, assembly, and regulation of multimolecular protein complexes (Neumann et al., 2010). Membrane order is primarily determined by membrane lipids’ tendency to phase separate (Sonnino and Prinetti, 2010). In Eukarya, membrane domains are thus specifically enriched in sphingolipids and cholesterol, which trigger liquid-liquid phase separation from the rest of the membrane (Wolf et al., 2001; Lindblom et al., 2006). However, other components and parameters have been proven essential for the lateral structuration of the membrane. For instance, specific proteins such as flotillins regulate membrane domain formation (García-Fernández et al., 2017), while the geometrical conformation of lipid polar head groups dictates their intermolecular interactions and lateral distribution (Hinz et al., 1991; Schütte et al., 2017). Although Bacteria do not synthesize cholesterol, membrane lateral organization has been recently expanded to this domain (LaRocca et al., 2013), and multidimensional structuration was thus suggested to be a fundamental feature of all biological membranes (Lingwood and Simons, 2010; López and Kolter, 2010).

The membrane lipids of archaea are, however, structurally divergent from those found in Bacteria and Eukarya. While the latter are typically composed of fatty-acyl ester linked to a glycerol backbone in *sn*-1,2 configuration, archaeal lipids are built upon isoprenoid cores that are ether linked to a *sn*-2,3 glycerol backbone (De Rosa and Gambacorta, 1988; Kamekura and Kates, 1999; Yoshinaga et al., 2015). Consequently, archaeal membranes are more stable and less permeable than those of Bacteria and Eukarya, enabling archaea to withstand a variety of environmental conditions, ranging from the mildest to the harshest known on Earth (Komatsu and Chong, 1998; Baba et al., 1999). Archaeal diether lipids are composed of C_15_ to C_25_ hydrocarbon chains that form bilayer membranes, whereas tetraether lipids contain C_40_ side chains linked to two glycerol moieties and thus form monolayer membranes. Archaeal core lipids display a diversity of structures, which includes mono- and dialkyl glycerol diethers [MGD and DGD (De Rosa et al., 1986)]; glycerol mono-, di-, and trialkyl glycerol tetraethers [GMGT, GDGT, and GTGT, respectively (Knappy et al., 2011)]; di- and tetraethers with hydroxylated, methylated, and unsaturated isoprenoid chains (Gambacorta et al., 1993; Nichols et al., 2004); and tetraethers with glycerol, butanetriol, and pentanetriol backbones (Becker et al., 2016). With phosphatidic- and glycosidic polar head groups deriving from typical sugars, amino acids, or combinations of both (Koga et al., 1993b), archaeal polar head group diversity does not fundamentally diverge from that of Bacteria and Eukarya. However, how these diverse archaeal lipids organize into functional membranes and whether lateral organization similar to that of eukaryotic and bacterial membranes exist in archaea remain elusive.

*Thermococcus barophilus* is a hyperthermophilic (optimal growth temperature 85°C) and piezophilic (optimal growth pressure 40 MPa) archaeon that synthesizes both diether and tetraether lipids (Cario et al., 2015). The presence of both types of lipids implies that parts of *T. barophilus* membrane are in the form of bilayers, whereas others are monolayered, thus delineating membrane domains reminiscent of the eukaryotic and bacterial membrane lateral structurations. Additionally, the insertion of apolar polyisoprenoids in the bilayer midplane was shown to trigger lipid phase separation (Salvador-Castell et al., 2020), suggesting that lateral organization is indeed possible in archaeal membranes. This model, based solely on the relative proportions of the different lipid classes in the membrane, does not account for lipid polar head groups whose charge, steric hindrance, geometry, polarity, and hydrophobicity are critical for lipid distribution and membrane surface properties, stability, impermeability, and functions (Hinz et al., 1985; Winter and Jeworrek, 2009; Bagatolli and Mouritsen, 2013). For instance, the average geometrical shape of lipids controls the propensity of these lipids to form specific phases, structures, and thus domains on small to large scales (Mouritsen, 2013). Resolving the entire diversity of archaeal lipids is thus of paramount importance to grasp their biological relevance, i.e., their physiological and adaptive functions, and to comprehend the membrane architecture and physicochemical properties in archaea.

Although essential to comprehend the membrane physiology, the structural diversity and distribution of archaeal lipids remain poorly characterized, partly because classic extraction procedures may lead to the preferential extraction of some classes of lipids over others (Huguet et al., 2010; Cario et al., 2015). The current data on archaeal lipids might thus not represent the real diversity in the original samples. Estimation of the lipid yield per cell indeed showed strong discrepancies with theoretical calculations of the total lipid content of different archaeal cells (Sinninghe Damsteì et al., 2002; Lipp et al., 2008; Schouten et al., 2012; Elling et al., 2014). For instance, intact polar lipid (IPL) extraction on *Methanothermobacter thermautotrophicus* yielded 0.038 to 0.26 fg IPL cell^–1^, whereas the theoretical lipid yield per cell for this rod-shaped archaeon (0.3 μm × 2.2 to 5.9 μm) is estimated to lie between 4.5 and 12.1 fg cell^–1^ (Yoshinaga et al., 2015). Similarly, the estimated lipid yield per cell for the coccus-shaped *Thermococcus kodakarensis* (1.1 to 1.3 μm) ranges from 7.5 to 10.8 fg cell^–1^, but the IPL extraction only yielded 0.38 to 1.61 fg cell^–1^ (Meador et al., 2014). In contrast, the IPL extraction on the much smaller rod-shaped archaeon *Nitrosopumilus maritimus* (0.2 μm × 0.5 to 0.9 μm) yielded similar lipid quantities than the theoretical estimation (0.9 to 1.9 fg cell^–1^ vs. 0.9 to 1.5 fg cell^–1^), respectively (Elling et al., 2014), which suggests that the archaeal lipid extraction efficiency might be impacted by physiological parameters, e.g., size and geometry of the cells, presence and characteristics of the cell envelope, as well as the lipids themselves, e.g., the nature of the polar head groups. Although cellular lipid contents were not estimated for *T. barophilus*, similar major extraction defects were also highlighted for this archaeon. The first and only characterization of the IPL signature of *T. barophilus* only reported PhosphatidylInositol-DialkylGlycerolDiether (PI)-DGD (Marteinsson et al., 1999). In agreement with this simple IPL composition, the acid methanolysis of the total lipid extract (TLE) yielded exclusively DGD (Marteinsson et al., 1999). However, the direct acid methanolysis of *T. barophilus* biomass revealed both a high proportion of tetraethers and a drastic bias of extraction and analytical procedures toward diether-based IPLs (Cario et al., 2015; Tourte et al., 2020b). Most of *T. barophilus* IPLs and polar head groups thus remain uncharacterized, impeding the understanding of its membrane physiology and organization.

In an effort to solve this missing IPL enigma, we comprehensively investigated *T. barophilus* IPL and polar head group compositions and examined its core lipid (CL) composition as a quality control of our methodology. We report the identification of up to 82 saturated and unsaturated IPLs, including the major PI-DGD, and the first characterization of several novel archaeal IPLs, notably phosphatidyl di-N-acetylhexosamine diethers and a tetraether bearing a peculiar derivative of glycosylated phosphatidylhexose. The unsuspectedly large IPL diversity of *T. barophilus* widens the Thermococcales lipid repertoire and contributes further refinements of the proposed membrane architecture in archaea.

## Materials and Methods

### Microorganism and Growth Conditions

*Thermococcus barophilus* strain MP was isolated from the 3,550-m deep Snake Pit hydrothermal vent on the Mid-Atlantic Ridge (Marteinsson et al., 1999). The strain was obtained from the UBOCC (Université de Bretagne Occidentale – type Culture Collection, France). The cultures were grown under strict anaerobiosis in a rich medium established for *Thermococcales* (Zeng et al., 2009), containing 3% *w*/*v* NaCl and 10 g L^–1^ elemental sulfur, at 85°C, pH 6.8, and atmospheric pressure. The medium was reduced by adding Na_2_S (0.1% *w*/*v* final) before inoculation. The growth was monitored by counting with a Thoma cell counting chamber (depth 0.01 mm) using a light microscope (life technologies EVOS XL Core, × 400). Under these conditions, cell concentrations of 2 × 10^8^ cells mL^–1^ were routinely achieved.

Cells of 1-L cultures in late exponential phase were recovered by centrifugation (4,000 × *g*, 45 min, 4°C) and rinsed twice with an isotonic saline solution (3% *w*/*v* NaCl). A significant amount of sulfur from the growth medium was recovered alongside cells, and the cellular dried mass was thus not estimated. The cell pellets were lyophilized overnight and kept at -80°C until lipid extraction.

### Intact Polar Lipid Extraction and Ultra-High Performance Liquid Chromatography-Electrospray Ionization-Tandem Mass Spectrometry Analysis

Archaeal IPLs are in most cases extracted using the modified Bligh and Dyer procedures (Bligh and Dyer, 1959), either with phosphate of trichloroacetic acid buffers (e.g., Evans et al., 2021). However, those were shown to be time consuming (due to long phase separation and/or acid neutralization), destructive, and incomplete for archaea such as *M. thermautotrophicus* (Dr. Philippe Schaeffer, unpublished results). We circumvent these issues by employing here a fast, soft-extraction method which does not require phase separation or acid neutralization and was already successfully applied to close relatives of *T. barophilus* (Tourte et al., 2020a). Briefly, dried cells were extracted with a monophasic mixture of methanol/dichloromethane/purified water (MeOH/DCM/H_2_O; 25:65:4; *v*/*v*/*v*) using a sonication probe for 15 min. After centrifugation (2,500 × *g*, 8 min), the supernatant was collected, and the extraction procedure was repeated twice. The supernatants were pooled, dried under a N_2_ stream, solubilized in MeOH/DCM (1:5; *v*/*v*; hereafter referred to as TLE), and kept at −20°C until analysis. A significant amount of sulfur from the growth medium was extracted alongside the archaeal lipids, and the total lipid dry mass was thus not estimated.

Intact polar lipid separation was first performed with a hydrophilic interaction liquid chromatography (HILIC) setting. IPLs were separated on a Waters Acquity UPLC BEH Amide 1.7 μm column (150 mm × 2.1 mm, Waters Corporation, Eschborn, Germany) maintained at 40°C by UHPLC using a Dionex UltiMate 3000RS UHPLC (ThermoFisher Scientific, Bremen, Germany) instrument equipped with an auto-injector and a Chromeleon chromatography manager software following the method described by Wörmer et al. (2013). Di- and tetraether IPLs were eluted in the same run with a flow rate of 0.4 mL min^–1^, using the following linear gradient with A [acetonitrile (ACN):DCM:formic acid (FA):ammonium hydroxide (NH_3(aq)_) (75:25:0.01:0.01, *v*/*v*/*v*/*v*)] and B [MeOH:H_2_O:FA:NH_3(aq)_ (50:50:0.4:0.4, *v*/*v*/*v*/*v*)]: 99% A (2.5 min isocratic) to 95% A for 1.5 min, then to 75% A for 18.5 min, and finally to 60% A for 4 min (1 min isocratic). Alternatively and when mentioned, IPL separation was performed with a reverse phase (RP) setting. Intact polar lipids (IPLs) were eluted on a Waters Acquity UPLC BEH C_18_ 1.7 μm column (150 mm × 2.1 mm, Waters Corporation, Eschborn, Germany) maintained at 65°C using the following linear gradient as described by Wörmer et al. (2013) with A [MeOH:H_2_O:FA:NH_3(aq)_ (85:15:0.04:0.1, *v*/*v*/*v*/*v*)] and B [propan-2-ol:MeOH:FA:NH_3(aq)_ (50:50:0.04:0.1, *v*/*v*/*v*/*v*)] at a flow rate of 0.4 mL min^–1^: 100% A (2 min isocratic) to 85% A for 0.1 min, then to 15% A for 18 min, and finally to 0% A for 0.1 min (8 min isocratic). One hundred percent A was eventually held isocratically for 7 min. Samples were thawed, dried under a N_2_ stream, and dissolved in either MeOH/DCM (1:9; *v*/*v*) or MeOH/DCM (9:1; *v*/*v*) for HILIC and RP separation, respectively. The injection volume was set to 10 μL.

Detection was achieved using a maXis quadrupole time-of-flight MS (Q-ToF-MS, Bruker Daltonics, Bremen, Germany) equipped with an ESI source operating in positive mode. The ESI source was also operated in negative mode, although this did not provide further information on the IPL composition. Only the conditions for the MS analysis in the positive mode are thus described here and were as follows: capillary voltage 4,500 V, nebulizer gas pressure 0.8 bar, drying gas (N_2_) flow 4 L min^–1^ at 200°C, and mass range *m/z* 100-2,000. MS calibration was performed by a tuning mixture solution (*m/z* 322.048, 622.029, 922.010, 1221.991, 1521.972, and 1821.952) introduced by loop injection near the end of a run and an internal lock mass [hexakis-(1H,1H,3H-tetrafluoro pentoxy)phosphazene, *m/z* 922.010] throughout the entire run. MS^2^ scans were automatically obtained in data-dependent mode by fragmentation of the three to ten most abundant ions at each MS scan using an intensity threshold of 3,000 counts and compounds exclusion after 5 fragmentations.

Mass spectra were visualized and analyzed using the Bruker Data Analysis software by comparing the parent molecular ion masses (occurring as H^+^, NH_4_^+^, or Na^+^ adducts) and the characteristic fragmentation patterns with the previously described ones (Yoshinaga et al., 2011; Lobasso et al., 2012; Meador et al., 2014). For quantification, 2 ng of a phosphatidylcholine C_21_-diacylglycerol (C_21_-PC) standard was added to the sample prior to injection. The response factors of bacterial mono- and digalactosyl diacylglycerols (MGDG and DGDG, respectively) relative to the injection standard C_21_-PC were used to approximate those of the detected IPLs. Calibration curves were established by injecting two times a standard solution consisting of C_21_-PC, MGDG, and DGDG in six different concentrations ranging from 0.001 to 30 ng μL^–1^. The detection was achieved only at concentrations higher than 0.1 ng μL^–1^. Under our analytical conditions, MGDG and DGDG showed molecular response factors of 0.58 and 0.21 relative to C_21_-PC, respectively. Different response factors are to be expected in ESI-MS, notably for lipids bearing distinct polar head groups (e.g., hexose vs. hexosamine), but the same response factors were applied for all IPLs bearing the same number of sugar residues in their polar head group, i.e., 0.58 for all mono- and 0.21 for all diglycosylated lipids, respectively. Intact polar lipid relative abundances were determined in the positive mode by integration of the peak area on the extracted ion mass chromatograms with a width of 0.02 Da corresponding to the protonated, ammoniated, and sodiated adducts, and subsequent correction using the corresponding response factor.

### Core Lipid Extraction and Ultra-High Performance Liquid Chromatography-Atmospheric Pressure Chemical Ionization-Mass Spectrometry Analysis

In order to exhaustively analyze the CL composition of *T. barophilus*, the polar head groups were removed by an acid methanolysis (1 N HCl in MeOH at 70°C for 16 h) of the biomass (total CLs) as described by Becker et al. (2013). The hydrolyzed lipids were extracted with a monophasic mixture of MeOH/DCM (1:5; *v*/*v*) using a sonication probe for 15 min. After centrifugation (2,500 × *g*, 8 min), the supernatant was collected in a separatory funnel and the extraction procedure was repeated twice. Core lipids (CLs) were partitioned into the organic phase following addition of Milli-Q water, and the aqueous phase was subsequently washed three times with an equal amount of DCM. The organic phases were collected, pooled, and subsequently washed three times with an equal amount of Milli-Q water. The solvent of the resulting CL extracts was evaporated under a N_2_ stream and the extracts were resolubilized in *n*-hexane/propan-2-ol (99.5:0.5; *v*/*v*). The same procedure was applied to the TLE (CLs from IPLs) to evaluate our IPL extraction method.

Core lipids were separated on two coupled Waters Acquity UPLC BEH Amide 1.7 μm columns (150 mm × 2.1 mm, Waters Corporation, Eschborn, Germany) maintained at 50°C using a Dionex UltiMate 3000RS UHPLC (ThermoFisher Scientific, Bremen, Germany) instrument equipped with an auto-injector and a Chromeleon chromatography manager software. The injection volume was set to 10 μL. Di- and tetraether CLs were eluted in the same run using a linear gradient with *n*-hexane and *n*-hexane/propan-2-ol (9:1; *v*/*v*) at a flow rate of 0.5 mL min^–1^, as described by Becker et al. (2013).

The detection was achieved using a maXis Q-ToF-MS (Bruker Daltonics, Bremen, Germany) equipped with an APCI source operating in positive mode. The conditions for the MS analyses were as in Becker et al. (2013): nebulizer gas pressure of 5 bar, corona discharge current of 3,500 nA, drying gas (N_2_) flow of 8 L min^–1^ at 160°C, vaporizer at 400°C, and mass range m/z of 150–2,000. The MS calibration and MS^2^ scans were performed as described above.

Mass spectra were visualized and analyzed on a Bruker Data Analysis software using parent molecular ion masses (occurring exclusively as H^+^ adducts) and characteristic fragmentation patterns (Hopmans et al., 2000). For quantification, 2 ng of a C_46_ analog of GTGT (C_46_-GTGT) was added to the sample prior injection. To determine the response factors of the detected core structures, calibration curves were established by injecting two times a standard solution consisting of C_46_-GTGT, GDGT with no cyclopentane ring (GDGT0), and DGD in 5 different concentrations ranging from 0.001 to 10 ng μL^–1^. GDGT0 was detected only at concentrations higher than 0.1 ng μL^–1^, whereas C_46_-GTGT and DGD were detected at all concentrations. Under our analytical conditions, DGD and GDGT0 showed relative response factors of 0.42 and 0.57 relative to C_46_-GTGT, respectively. In the absence of a measured response factor for the different archaeal core lipids, we assumed the same response factor as DGD for all diethers and that of GDGT0 for all tetraethers. CL relative abundances were determined by integration of the peak area on the extracted ion mass chromatograms with 0.1 Da width corresponding to the protonated adducts and the subsequent correction by the corresponding response factor.

### Isolation of *Thermococcus barophilus* Major Intact Polar Lipids

In order to further characterize and validate the structural diversity of *T. barophilus* lipids, its major IPLs were isolated using the aforementioned HILIC UHPLC method. Fifty percent of the TLEs were dried under a N_2_ stream and resolubilized in 10 μL of MeOH/DCM (1:9; *v*/*v*) for injection. The collecting vials were placed at the exit of the chromatography column, and 7 fractions corresponding to *T. barophilus* well-separated major IPLs were manually collected (F1, 11.5–12.5 min; F2, 13.3–14.0 min; F3, 14.5–15.5 min; F4, 15.7–16.4 min; F5, 17.9–18.2 min; F6, 18.4–19.0 min; F7, 23.0–27.0 min). The collected fractions were dried under a N_2_ stream, resolubilized in 100 μL of MeOH/DCM (9:1; *v*/*v*), and their purity was assayed using the aforementioned RP UHPLC-MS method. The injection volume was set to 10 μL. The detection was achieved as described in the previous section.

### Intact Polar Lipid Cleavage and Ultra-High Performance Liquid Chromatography-Electrospray Ionization-Tandem-Triple Quadrupole-Mass Spectrometry Analysis of the Hexose-Based Polar Head Groups

In order to characterize the hexose-based polar head groups of *T. barophilus*, both the biomass and individual IPL fractions were cleaved by acid hydrolysis [30% trifluoroacetic acid (TFA) in H_2_O at 70°C for 16 h] to release the monosaccharide(s) from IPLs. The reaction was stopped by drying the sample under a stream of N_2_ and the remaining TFA was removed by washing three times with DCM. The hydrolysates were solubilized in ACN/H_2_O (95:5, *v*/*v*), and the CLs were extracted upon addition of hexane while monosaccharides remained in the ACN/H_2_O phase.

Core lipids were separated and analyzed using the aforementioned UHPLC-APCI-MS system.

Monosaccharides were separated on a Waters Acquity UPLC BEH Amide 1.7 μm column (150 mm × 2.1 mm, Waters Corporation, Eschborn, Germany) maintained at 60°C using a Dionex UltiMate 3000RS UHPLC (ThermoFisher Scientific, Bremen, Germany). Samples were dissolved in ACN/H_2_O (95:5, *v*/*v*) and the injection volume was set to 10 μL. All hexose-based polar head groups were eluted in the same run using the linear gradient described by Lowenthal et al. (2015) with A [0.1% NH_3(aq)_ in H_2_O] and B [0.1% NH_3(aq)_ in ACN] at a flow rate of 0.2 mL min^–1^: 5% A (3 min isocratic) to 10% A for 22 min, then to 40% A for 3 min (7 min isocratic), and finally to 5% A for 2 min. Detection was achieved by a scheduled multiple reaction monitoring (sMRM) with a mass resolution of 0.1 Da on a QTRAP 4500 QQQ MS (ABSciEX, Darmstadt, Germany) equipped with an ESI source operating in positive mode. The source conditions were as follows: curtain gas (CUR) pressure of 30 psi, ion source gas 1 (GS1) pressure of 40 psi, ion source gas 2 (GS2) pressure of 30 psi, ion spray (IS) voltage of 4,500 V, and capillary temperature (TEM) of 400°C. The MRM method was established by direct infusion of 14 carbohydrates (Supplementary Table 1) and consisted of 20 different transitions, with two transitions for each carbohydrate type.

The different sugar-based head groups were quantified by external calibration. Linear calibration curves were established for a wide variety of sugar derivatives by injecting two times standard solutions containing β-D-allopyranose (Alp), β-D-fructopyranose (Fru), α-D-glucopyranose (Glc), β-D-galactopyranose (Gal), α-D-mannopyranose (Man), β-D-xylofuranose (Xyl), α-L-arabinopyranose (Ara), α-D-lyxopyranose (Lyx), D-glucosamine (GlcNH_2_), N-acetyl-D-glucosamine (GlcNAc), myo-inositol (Ino), and D-saccharose (Sac) in 12 different concentrations ranging from 0.005 to 1,000 μM. Alp and Fru and Xyl and Ara peaks could not be distinguished and were integrated as two single peaks (Alp/Fru and Xyl/Ara, respectively). Every standard was detected in concentration as low as 0.05 μM, with the exception of Sac which was not identified below 5 μM.

### ExtractIon-Free Analysis *via* Matrix-Assisted Laser Desorption/Ionization Fourier Transform Ion Cyclotron Resonance Mass Spectrometry

To further investigate the IPL diversity of *T. barophilus*, and particularly that of tetraether-based lipids, the MALDI-FT-ICR-MS was used directly on the biomass.

*Thermococcus barophilus* dried cell pellets were resuspended in 1 mL of Milli-Q water and centrifuged (500 × *g*, 1 min) to remove as much sulfur as possible. Supernatants were then decanted by centrifugation (15,000 × *g*, 15 min) and cells were resuspended in Milli-Q water. Ten milligrams of 2,3-dihydroxybenzoic acid (DHB) was dissolved in 1 mL of H_2_O/ACN (3:7, *v*/*v*) containing 1% of TFA and used as matrix. The cell suspension and the matrix solution were mixed (1:1; *v*/*v*) and 1 μL was spotted and evaporated on a ground steel MALDI target plate.

The analysis was carried out on a 7T solariX XR FT-ICR-MS coupled to a DUAL source with a Smartbeam II laser (Bruker Daltonics, Bremen, Germany) operating at 355 nm. The FT-ICR-MS was operated in positive serial mode, with data being acquired over the mass range *m*/*z* 600-3,000. The instrument settings were optimized for larger molecules (*m*/*z* > 1,000). Each scan was generated by accumulating the ions of 25 laser shots at 40% laser power and a frequency of 200 Hz. External calibration was performed with a standard peptide mixture (Bruker Daltonics). An internal lock mass calibration was applied using the sodiated adduct of PI-GDGT0-PI (*m/z* = 1808.343). Using our improved parameters, a mass resolution of *ca* 0.01 Da was achieved for the most abundant compound (*m/z* = 917.686).

## Results

### *Thermococcus barophilus* Exhibits a Diverse Membrane Lipid Composition

**FIGURE 1:**
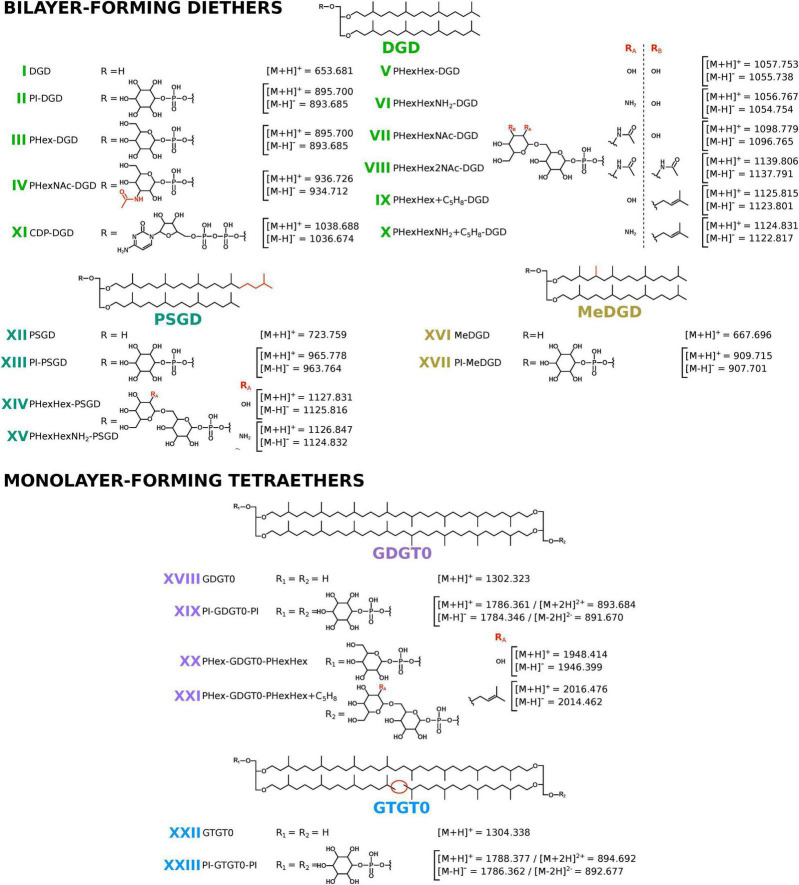
Core and intact polar lipids of *Thermococcus barophilus*. Short-hand nomenclature is indicated. The protonated, ammoniated, and sodiated adducts and only the deprotonated adducts were detected in positive and negative ion modes, respectively. Only the protonated and deprotonated ions are represented in the figure. Core structures: diphytanyl glycerol diethers (DGD; light green; I to XI), phytanylsesterterpanyl glycerol diethers (PSGD; dark green; XII to XV), DGD bearing an additional methylation (MeDGD; yellow; XVI and XVII), glycerol dibiphytanyl (or dialkyl) glycerol tetraethers with no cyclopentane ring (GDGT0; purple; XVIII to XXI), and glycerol biphytanyl diphytanyl (or trialkyl) glycerol tetraethers with no cyclopentane ring (GTGT0; blue; XXII and XXIII). Note that II, III, IV, VI, and XI were detected with up to 8 unsaturations, whereas V, VII, IX, and X were detected with up to 6 unsaturations. No unsaturation was detected in the other core structures. Unsaturations are not represented. Polar head groups: phosphatidylinositol (PI; II, XIII, XVII, XIX, and XXIII), phosphatidylhexose (PHex; III, XX, and XXI), phosphatidyl-N-acetylhexosamine (PHexNAc; IV), glycosylated phosphatidylhexose (PHexHex; V, XIV, and XX), ammoniated PHexHex (PHexHexNH_2_; VI and XV), one and two N-acetylated PHexHex (PHexHexNAc and PHexHex2NAc; VII and VIII), PHexHex and PHexHexNH_2_ bearing an additional mass of 68 (PHexHex + C_5_H_8_ and PHexHexNH_2_ + C_5_H_8_; IX and XXI, and X), and cytidine diphosphate (CDP; XI). Positions of additional methylation and of additional groups on the polar head groups are drawn arbitrarily in the figure.

Our extracts analyzed with the UHPLC-MS procedure yielded very low cellular lipid content (0.12 fg cell^–1^, as calculated from cell counts and lipid abundance). The combined analyses of Kraft (2013) the TLE and Nickels et al. (2019) the polar head groups and core structures separated from purified major lipids nevertheless revealed a diverse membrane composition for *T. barophilus* (for structures, refer to Figure 1). All IPLs identified in *T. barophilus* TLE showed ion diagnostic of archaeal phosphoglycolipids (for instance, ions at *m/z* = 733.6 and 453.3; Supplementary Figure 1), whereas no glycolipids were detected.

**FIGURE 2:**
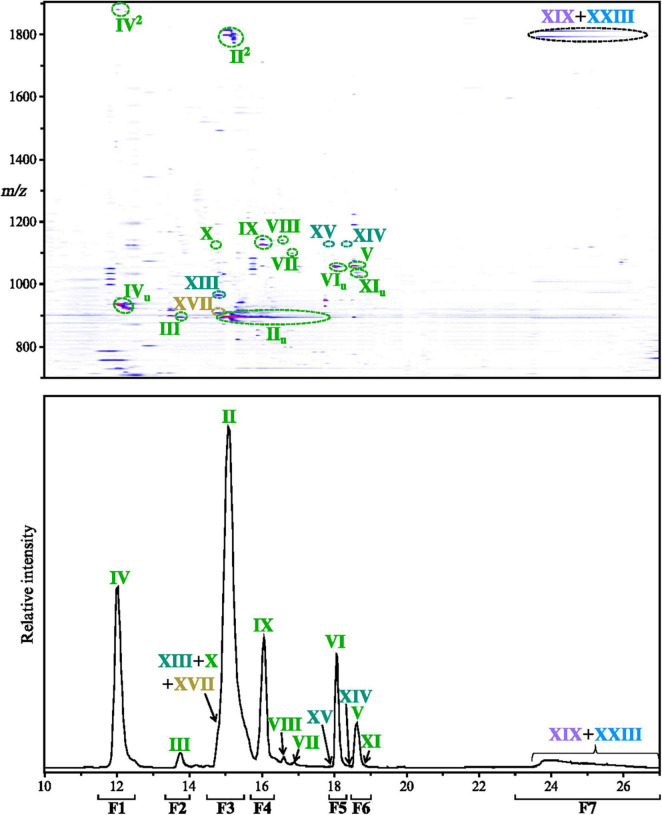
*Thermococcus barophilus* exhibits a large diversity of intact polar lipids. Intact polar lipids were detected in positive and negative ion modes. As no additional intact polar lipid (IPL) could be identified in the negative ion mode, only the density map and chromatogram obtained in positive ion mode are displayed (zoom in the 10–27 min, *m/z* 700–1900 window). Compounds detected with unsaturations and/or diadducts are marked with _*u*_ and ^2^, respectively. The ultra-high performance liquid chromatography (UHPLC) chromatogram was drawn by extracting the following protonated ion masses with a mass deviation of ± 0.02 Da: 893.68, 894.70, 895.70, 909.72, 936.73, 965.78, 1038.69, 1056.77, 1057.75, 1098.78, 1124.83, 1125.82, 1126.85, 1127.83, 1139.81, 1786.36, and 1788.38. Refer to Figure 1, Table 1, and Supplementary Figures 1–9 for lipid structures and their respective molecular masses. F1–F7 delineate the time range corresponding to each fraction collected to confirm the structures of the identified lipids (refer to the Methods section).

The TLE of *T. barophilus* appeared to be dominated by compound II (Figure 2 and Table 1), whose molecular mass ([M + H]^+^ at *m/z* = 895.704) and fragmentation pattern {for instance, ions at *m/z* = 615.4 and 733.6 can be attributed to the IPL after the loss of one C_20_ isoprenoid chain ([M + H]^+^ minus 280.3) and to the IPL after the loss of one hexose moiety ([M + H]^+^ minus 162.1)}, respectively (Supplementary Figure 2) corresponded to a DGD bearing a phosphatidylhexose head group. The molecular mass and fragmentation pattern of the minor compound III (Supplementary Figure 2) appeared identical to compound II but the difference in retention times (13.7 vs. 15.0 min for compounds III and II, respectively) indicated distinct hexose isomers as polar head groups of II and III. The analysis of the purified fractions revealed 100% of Ino and 98% of DGD for fraction F3 (98% of compound II) and 100% of Glc and 100% of DGD for fraction F2 (containing only III; Table 2), respectively. This confirmed II to be a PI-DGD and III its Glc isomer PGlc-DGD. Compound IV was another major IPL of *T. barophilus* TLE (Figure 2 and Table 1). Its molecular mass ([M + H]^+^ at *m/z* = 936.713); retention time (12.2 min); fragmentation pattern {for instance, ions at *m/z* = 138.1 and 204.1 can be attributed to the IPL head group after the loss of three hydroxyl groups ([M + H]^+^ = 17 each) and one primary alcohol ([M + H]^+^ = 32), and of one hydroxyl group, respectively; Supplementary Figure 2}; and the presence of 52% of Glc and 48% of GlcNAc and of 100% of DGD in fraction F1 (containing only IV) allowed to identify as a PGlcNAc-DGD (Table 2). The fragmentation pattern of IV did not allow to determine the exact position of the N-acetylation.

**TABLE 1:** Intact polar lipid structures and lipid composition (absolute quantity, cellular abundance, and molar relative%) of *Thermococcus barophilus*.

Lipid	Core	Head group	Acronym	Chemical formula	Theoretical [M + H]^+^	Unsaturations	HPLC-ESI-MS					MALDI FT-ICR-MS
							RT (min)	Detected [M + H]^+^	Absolute quantity (ng)*^a^*	Cellular abundance (fg cell^–1^)*^b^*	Molar relative%*^c^*	Detected [M + Na]^+^
II	DGD	Phosphatidyl inositol	PI-DGD	C_49_H_99_O_11_P	895.700	0–8	15.0	895.7042	21500	0.11	90.0	917.686
III	DGD	Phosphatidyl glucose	PGlc-DGD	C_49_H_99_O_11_P	895.700	0–8	13.7	895.6879	115	5.8 × 10^–4^	Traces	917.686
IV	DGD	Phosphatidyl N-acetylglucosamine	PGlcNAc-DGD	C_51_H_103_O_11_P	936.726	0–8	12.2	936.7133	1175	5.9 × 10^–3^	4.9	ND
V	DGD	Phosphatidyl glucose + glucose	PGlcGlc-DGD	C_55_H_109_O_16_P	1057.753	0–6	18.6	1057.7536	230	1.2 × 10^–3^	Traces	1079.741
VI	DGD	Phosphatidyl glucose + hexosamine	PGlcHexNH_2_-DGD	C_55_H_110_NO_15_P	1056.769	0–8	18.0	1056.7736	305	1.5 × 10^–3^	1.3	ND
VII	DGD	Phosphatidyl hexose + N-acetylhexosamine	PHexHexNAc-DGD	C_57_H_112_NO_16_P	1098.779	0–6	16.9	1098.7805	10	5.0 × 10^–5^	Traces	ND
VIII	DGD	Phosphatidyl hexose + di-N-acetylhexosamine	PHexHex2NAc-DGD	C_59_H_115_N_2_O_16_P	1139.806	0	16.6	1139.8062	20	1.0 × 10^–4^	Traces	ND
IX	DGD	Phosphatidyl glucose + hexose + C_5_H_8_	PGlcHex + C_5_H_8_-DGD	C_60_H_117_O_16_P	1125.815	0–6	16.0	1125.8164	370	1.9 × 10^–3^	1.5	1147.804
X	DGD	Phosphatidyl glucose + hexosamine + C_5_H_8_	PGlcHexNH_2_ + C_5_H_8_-DGD	C_60_H_118_NO_15_P	1124.831	0–6	14.7	1124.8270	1	5.0 × 10^–6^	Traces	ND
XI	DGD	Cytidine diphosphate	CDP-DGD	C_52_H_101_N_3_O_13_P_2_	1038.688	0–8	18.7	1038.6887	10	5.0 × 10^–5^	Traces	ND
XIII	PSGD	Phosphatidyl inositol	PI-PSGD	C_54_H_109_O_11_P	965.778	0	14.8	965.7780	65	3.3 × 10^–4^	Traces	ND
XIV	PSGD	Phosphatidyl glucose + glucose	PGlcGlc-PSGD	C_60_H_119_O_16_P	1127.831	0	18.5	1127.8274	4	2.0 × 10^–5^	Traces	ND
XV	PSGD	Phosphatidyl glucose + hexosamine	PGlcHexNH_2_-PSGD	C_60_H_120_NO_15_P	1126.847	0	17.8	1126.8424	3	1.5 × 10^–5^	Traces	ND
XVII	MeDGD	Phosphatidyl inositol	PI-MeDGD	C_50_H_101_O_11_P	909.715	0	14.9	909.6897	NQ	NQ	NQ	ND
XIX	GDGT	Diphosphatidyl inositol	PI-GDGT0-PI	C_98_H_194_O_22_P_2_	1786.361	0	23.4-26.6	1786.3595	100	5.0 × 10^–4^	Traces	1808.354
XX	GDGT	Phosphatidyl hexose + phosphatidyl hexose + hexose	PHex-GDGT0-PHexHex	C_104_H_204_O_27_P_2_	1948.414	0	ND	ND	ND	ND	ND	1970.412
XXI	GDGT	Phosphatidyl hexose + phosphatidyl hexose + hexose + C_5_H_8_	PHex-GDGT0-PHexHex + C_5_H_8_	C_109_H_213_O_27_P_2_	2016.476	0	ND	ND	ND	ND	ND	2038.486
XXIII	GTGT	Diphosphatidyl inositol	PI-GTGT0-PI	C_98_H_196_O_22_P_2_	1788.377	0	23.4-26.6	1788.3763	NQ	NQ	NQ	1810.361

*Traces, < 1%. ND, not detected; NQ, not quantified; DGD, dialkyl glycerol diethers; PSGD, phytalsesterterpanyl glycerol diethers; MeDGD, methylated DGD; GDGT, glycerol dialkyl glycerol tetraethers; GTGT, glycerol trialkyl glycerol tetraethers. ^a^2 ng of the internal standard C_21_-PC was injected to quantify the identified IPL. Quantities account for protonated, ammoniated and sodiated adducts of saturated IPLs in ESI positive mode and were calculated assuming a response factor of 0.58 for monoglycosidic IPLs and 0.21 for diglycosidic IPLs relative to the internal standard C_21_-PC (refer to methods). ^b^Calculated with an average cell number of 2.0 × 10^8^ cell mL^–1^ (refer to methods). ^c^Molar relative proportions were calculated from each IPL quantity weighted by their respective molar mass.*

**TABLE 2:** Characteristics and distribution of the core structures and the polar head groups of purified major lipids of *Thermococcus barophilus*.

Fraction	Time range (min)	Expected IPL	Detected IPL (%)^a^	Core structure^b^					Polar head^c^								
				DGD I	PSGD XII	MeDGD XVI	GDGT0 XVIII	GTGT0 XXII	Pent	All/Fru	Man	Gal	Glc	Ino	2Hex	GlcNAc	Acido Hex
F1	11.5–12.5	**IV**	**IV** (100)	100	ND	ND	ND	ND	ND	ND	ND	ND	58	ND	ND	42	ND
F2	13.3–14.0	**III**	**III** (100)	100	ND	ND	ND	ND	ND	ND	ND	ND	100	ND	ND	ND	ND
F3	14.5–15.5	**II** + **X** + **XIII** + **XVII**	**II**(98) + **XIII**(2) + **XVII**(traces) + **X**(traces)	98	2	ND	ND	ND	ND	ND	ND	ND	ND	100	ND	ND	ND
F4	15.7–16.4	**IX**	**IX**(93) + **II**(7) + **V**(traces) + **XIII**(traces)	100	Traces	ND	ND	ND	ND	ND	ND	ND	100	ND	ND	ND	ND
F5	17.9–18.2	**VI** + **XV**	**VI**(95) + **IX**(3) + **II**(2) + **XV**(traces)	100	Traces	ND	ND	ND	ND	ND	ND	ND	100	ND	ND	ND	ND
F6	18.4–19.0	**V** + **XI** + **XIV**	**V**(98) + **II**(1) + **VI**(1) + **XIV**(traces)	100	Traces	ND	ND	ND	ND	ND	ND	ND	100	ND	ND	ND	ND
F7	23.0–27.0	**XIX** + **XXIII**	**I** (100)	2	ND	ND	95	3	ND	ND	ND	ND	ND	43	57	ND	ND

*Traces, < 1%. ND, not detected; DGD, dialkyl glycerol diethers; PSGD, phytanylsesterterpanyl glycerol diethers; MeDGD, methylated DGD; GDGT0, glycerol dialkyl glycerol tetraethers with no cyclopentane ring; GTGT0, glycerol trialkyl glycerol tetraethers with no cyclopentane ring; Pent, β-D-xylofuranose, α-L-arabinopyranose and α-D-lyxopyranose; All/Fru, β-D-allopyranose and β-D-fructopyranose; Man, D-mannopyranose; Gal, β-D-galactopyranose; Glc, α-D-glucopyranose; Ino, myo-inositol; 2Hex, dihexoses; GlcNAc, α-N-acetyl-D-glucosamine; AcidoHex, D-galacturonic acid and D-glucuronic acid, D-galactosamine. ^a^Relative proportions account for protonated, ammoniated and sodiated adducts of saturated IPLs in ESI positive mode and were calculated assuming a response factor of 0.58 for monoglycosidic IPLs and 0.21 for diglycosidic IPLs relative to the internal standard C_21_-PC (refer to methods). ^b^Relative proportions account for protonated adducts in APCI positive mode and were calculated assuming a response factor of 0.42 for diethers and 0.57 for tetraethers relative to the internal standard C_46_-GTGT, or of 0.74 for diethers relative to tetraethers (refer to methods). ^c^Relative proportions were calculated assuming the response factors determined for each sugar using a standard solution (refer to methods).*

Compounds XIII and XVII had retention times almost identical to compound II (14.8 and 14.9 min, respectively; Figure 2), but higher molecular masses ([M + H]^+^ at *m/z* = 965.778 and 909.690, respectively), and fragmentation patterns that suggested these compounds to be a phytanylsesterterpanyl glycerol diether (PSGD) and a methylated DGD (MeDGD)-bearing phosphatidylhexose head groups (for instance, ions at *m/z* = 723.8 and 649.7 can be attributed to PSGD and MeDGD core lipids, respectively; Supplementary Figure 3). The analysis of fraction F3 containing compounds II, XIII, and XVII showed exclusively Ino and 98% of DGD and 2% of PSGD (Table 2). The structures of PI-DGD II and PI-PSGD XIII were thus confirmed while compound XVII was only suggested to correspond to PI-MeDGD.

Compounds V and VI, which differed from one another by slightly less than one mass unit ([M + H]^+^ at *m/z* = 1057.754 and 1056.774, respectively) and showed similar fragmentation patterns {for instance, ions at *m/z* = 777.4 and 776.5 can be attributed to phosphatidyldihexose and its hexosamine derivative after the loss of one C_20_ isoprenoid chain ([M + H]^+^ minus 280.3), respectively; Supplementary Figure 4}, were identified as DGD bearing a glycosylated phosphatidylhexose head group and its hexosamine derivative in which one hydroxyl group is replaced by an amino group, respectively. The acid hydrolysis of the fractions containing V and VI (F6 and F5, respectively) yielded only Glc and DGD with traces of PSGD (Table 2). The presence of only Glc and the absence of disaccharides in both F5 and F6 suggested that the hydrolytic conditions were adequate to cleave off the glycosidic bond between the two sugar moieties, and that the structure of V was probably PGlcGlc-DGD. No hexosamine was detected in F5, impeding further characterization of compound VI, which we therefore tentatively assigned to PGlcHexNH_2_-DGD. Similarly to V and VI, compounds XIV and XV also differed from one another by slightly less than one mass unit ([M + H]^+^ at *m/z* = 1127.827 and 1126.842, respectively) and were present in fractions F6 and F5, respectively. Their molecular masses shifted upward by 70 mass units compared with PGlcGlc-DGD V and PGlcHexNH_2_-DGD VI, retention times (18.5 and 17.8 min, respectively), fragmentation patterns {for instance, ions at *m/z* = 685.5 can be attributed to PHex-PSGD after the loss of one C_20_ isoprenoid chain ([M + H]^+^ minus 280.3), Supplementary Figure 5}, and the presence of traces of PSGD in F6 and F5 (Table 2) suggested compounds XIV and XV to correspond to PGlcGlc-PSGD and PGlcHexNH_2_-PSGD, respectively. Similarly to compound VI, compounds VII, VIII, IX, and X ([M + H]^+^ at *m/z* = 1098.781, 1139.806, 1125.815, and 1124.831, respectively) could be associated with derivatives of PGlcGlc-DGD V (Table 1 and Supplementary Figures 4, 6, 7). The comparison of the fragmentation patterns of VII and VIII with those of PGlcNAc-DGD IV and PGlcGlc-DGD V, and notably the detection of ions at *m/z* = 138.1 and 818.4 {corresponding to the IPL head group after the loss of three hydroxyl groups and one primary alcohol and to the IPL after the loss of one C_20_ isoprenoid chain ([M + H]^+^ = 280.3, respectively)} and at 245.1 and 859.5 (corresponding to the IPL head group after the loss of one hydroxyl group and to the IPL after the loss of one C_20_ isoprenoid chain, respectively; Supplementary Figures 4, 6), suggested the former to correspond to DGDs bearing mono- (PHexHexNAc-DGD VII) and di-N-acetylated glycosylated phosphatidylhexose (PHexHex2NAc-DGD VIII), respectively. While the exact nature of the sugar moieties and the positions of the N-acetylations could not be further resolved, it is worth noting that these compounds as well as other nitrogen-containing IPLs and their fragments strictly follow the nitrogen rule, which can be of great help while trying to decipher novel IPL mass spectra. Compounds IX and X showed molecular masses ([M + H]^+^ at *m/z* = 1125.816 and 1124.827, respectively) shifted upward by 68 mass units compared with PGlcGlc-DGD V and PGlcHexNH_2_-DGD VI, respectively, and displayed similar fragmentation patterns {for instance, ions at *m/z* = 845.5 and 844.5 can both be attributed to IPLs after the loss of one C_20_ isoprenoid chain ([M + H]^+^ minus 280.3; Supplementary Figures 4, 7)}. Although the nature of this increase in 68 Da could not be determined, we suggested it to be an isoprene unit (C_5_H_8_). The acid hydrolysis of fraction F4 (93% of IX) released a majority of DGD and exclusively Glc (Table 2), and compound IX was thus assigned the partially resolved structure PGlcHex + C_5_H_8_-DGD. Compound X was detected in very low amount (5.0 × 10^–6^ fg cell^–1^; Table 1), which hinders its complete characterization. Although no Glc was detected in F3 containing X, its similarity to both PGlcHexNH_2_-DGD VI and PGlcHex + C_5_H_8_-DGD IX (Supplementary Figures 4, 7) suggested it to correspond to PGlcHexNH_2_ + C_5_H_8_. Other minor compounds with masses shifted upward by either 68 or 70 Da were detected, e.g., a compound at 15.8 min with [M + H]^+^ at *m/z* = 1195.886 could correspond to PGlcGlc-PSGD with 68 additional Da, i.e., PGlcGlc + C_5_H_8_-PSGD. Their low abundances and the absence of MS^2^ spectra, however, prevented to resolve their exact structures.

Apart from the ions typical of archaeal phospholipids (Supplementary Figure 1), compound XI ([M + H]^+^ at *m/z* = 1038.689) showed a fragmentation pattern completely distinct from the IPLs described above which suggested a head group distinct from typical hexoses {for instance, ions at *m/z* = 324.1 and 758.4 can be attributed to a cytidine monophosphate and to the IPL after the loss of one C_20_ isoprenoid chain ([M + H]^+^ minus 280.3, respectively; Supplementary Figure 8)}. Compound XI could indeed be attributed to a DGD bearing a diphosphatidyl cytidine head group, i.e., the first intermediate in the pathway for polar head group fixation (Morii et al., 2000). The lack of relevant standard and the low quantities of XI prevented further elucidation of its structure by the acid hydrolysis of fraction F6 (Table 2).

In addition to their fully saturated forms, minute amounts of PI-DGD II, PGlc-DGD III, PGlcNAc-DGD IV, PGlcHexNH_2_-DGD VI, and CDP-DGD XI were detected with one to eight unsaturations, whereas PGlcGlc- V, PHexHexNAc- VII, PGlcHex + C_5_H_8_- IX, and PHexHexNH_2_ + C_5_H_8_-DGD X were detected with one to six unsaturations (Figure 2). Only the fully saturated forms were detected for the other diethers, namely PHexHex2NAc-DGD VIII, PI-PSGD XIII, PGlcGlc-PSGD XIV, PGlcHexNH_2–_PSGD XV, and PI-MeDGD XVII.

As various glucose derivatives were detected, we looked for putative IPLs bearing similar and additional combinations of NH_2_, NAc, and/or C_5_H_8_ groups, but none were detected. Similarly, glycolipids and phospholipids regularly found in archaea, e.g., lipids bearing phosphatidylcholine (PC), phosphatidylserine (PS), and phosphatidylethanolamine (PE), were searched for but not detected in *T. barophilus*.

The very few tetraether-based IPLs detected in *T. barophilus* TLE all gather in a poorly resolved broad peak (Figure 2). The major component of this peak was compound XIX, whose molecular mass ([M + H]^+^ at *m/z* = 1786.360), fragmentation pattern {for instance, ions at *m/z* = 731.6 and 1544.3 can be attributed to the [M + 2H]^2+^ adduct of the IPL after the loss of two hexose moieties ([M + H]^+^ minus twice 162.1) and after the loss of one phosphatidylhexose ([M + H]^+^minus 242.1), respectively; Supplementary Figure 9}, and the acid hydrolysis of F7 (95% of GDGT0, 43% of Ino; Table 2) identified it as PI-GDGT0-PI. No fragmentation pattern was obtained for compound XXIII, but its molecular mass ([M + H]^+^ at *m/z* = 1788.376) shifted upward by two mass units compared to XIX and the presence of 3% of GTGT0 in F7 suggested that it might correspond to PI-GTGT0-PI (Table 2; see further details in Supplementary Text). F7 also contained 57% of a compound with the same fragmentation pattern as the Sac standard but with a slightly distinct retention time, which thus suggested it to be another disaccharide (hereafter referred to as 2Hex). The abundance of di-hexose in fraction F7 and the absence of tetraether-based IPLs with a 2Hex polar head group in UHPLC-ESI-MS suggested that this fraction might contain other, unresolved tetraether-based IPLs.

In addition to this set of IPLs, we detected a series of polyprenyl derivatives in *T. barophilus* TLE using our RP UHPLC method (Supplementary Figure 11). These compounds represent a large family of membrane-bound polyisoprenoids known as major lipid carriers for membrane protein glycosylation in all three domains of life (Hartley and Imperiali, 2012). Such compounds have been identified in a wide variety of archaea, in which the sugar residue can be attached to either alcohol, mono-, or diphosphate end groups of polyprenyls with six to 14 isoprene units (Salvador-Castell et al., 2019). Here, the polyprenyl derivatives comprising 10 to 12 isoprene units and 1 to 5 unsaturations with only monophosphate head groups were identified (Supplementary Figure 11).

### PI-DGD Dominates the Total Lipid Extract of *Thermococcus barophilus*

The IPLs identified in *T. barophilus* TLE were quantified considering response factors of 0.58 for monoglycosidic (II, III, IV, XI, XIII, and XVII) and 0.21 for diglycosidic IPLs (V, VI, VII, VIII, IX, X, XIV, XV, XIX, and XXIII) relative to the internal standard C_21_-PC. Since unsaturated IPLs were detected in minute amounts, only the saturated forms were quantified. *T. barophilus* TLE (0.12 fg cell^–1^) was overwhelmingly dominated by PI-DGD II (91%, 0.11 fg cell^–1^; Table 1), with a few other major IPLs, i.e., PGlcNAc-DGD IV, PGlcHexNH_2_-DGD VI, PGlcHex + C_5_H_8_-DGD IX, and PGlcGlc-DGD V (*ca.* 8%, 1.05 × 10^–2^ fg cell^–1^). The remaining minor IPLs detected in trace amounts or in too low abundances to be quantified represented only 6.25 × 10^–3^ fg cell^–1^ (*ca.* 1% of the TLE mass). DGD-based IPLs represented *ca.* 99% of *T. barophilus* TLE, whereas tetraether-based IPLs were only recovered in trace amounts (Table 1).

**FIGURE 3:**
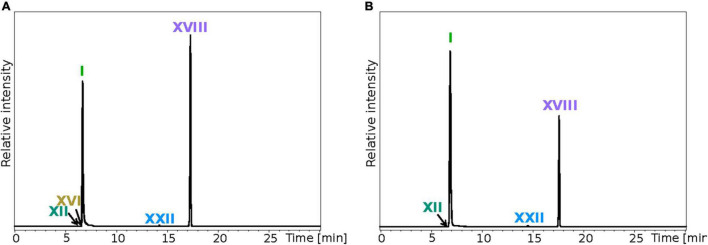
Analysis of the core lipids (CLs) highlights a significant discrepancy between *T. barophilus* total and extracted lipids. Total CLs **(A)** and CLs from IPLs **(B)** were recovered after methanolysis of the biomass and the total lipid extract (TLE), respectively. Direct methanolysis and intact polar lipid extraction were both performed on the same amount of biomass. UHPLC chromatograms were drawn in positive mode by extracting the following protonated ions with a mass deviation of ± 0.1 Da: 653.68, 667.70, 723.76, 743.71, 1302.32, and 1304.34. Refer to Figure 1 for lipid structures.

**FIGURE 4:**
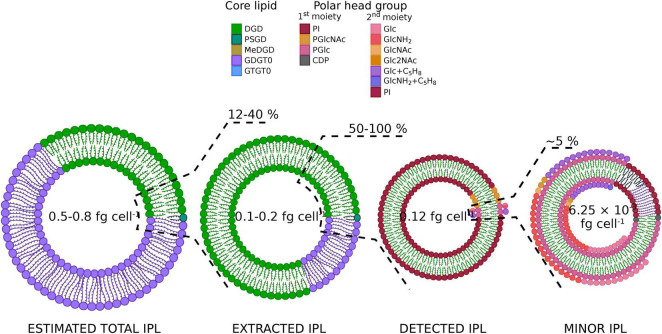
The vast majority of *Thermococcus barophilus*’ lipidome remains inaccessible. Pie chart representations of *T. barophilus* lipids at each extraction step. The direct acid methanolysis of *T. barophilus* biomass yielded its total core lipids content, which contained *ca*. 30% of diethers (DGD, green; PSGD, dark green; MeDGD, dark yellow) and 70% of tetraethers (GDGT0, purple; GTGT0, blue; Table 3). Considering putative low and high mass polar head groups found in archaea, the direct acid methanolysis of the biomass yielded an experimentally derived theoretical lipid content of 0.5 to 0.8 fg of lipids cell^– 1^. An acid methanolysis of the TLE allowed the determination of the extraction yield on *T. barophilus* (0.1 to 0.2 fg cell^– 1^ considering putative low and high mass polar head groups, *ca.* 12–40% of the biomass’ total lipids) and of the diether and tetraether distribution in extracted lipids (80/20; Table 3). Although part of *T. barophilus* IPLs is indeed extracted, the majority of its IPLs, and especially tetraether-based ones, remain resistant to extraction. Eighteen IPLs representing 50–100% of these extracted lipids (0.12 fg cell^– 1^) were identified with UHPLC-MS (Table 1). The putative discrepancy between extracted and identified IPLs highlights only a partial detection of *T. barophilus* IPLs with our UHPLC-MS system. Complex IPLs with at least two sugar residues (1*^st^* and 2*^nd^* polar head moieties) were recovered with an even lower yield, i.e., 6.25 × 10^– 3^ fg cell^– 1^, *ca.* 5% of the IPLs identified (Table 1), thus showing that peculiar polar head groups might be the main reason for the detection defect observed in *T. barophilus.*

To evaluate the efficiency of our extraction and analysis protocols, we performed several calculations to approximate a putative total IPL composition of TLE and of the original biomass. The acid methanolysis of the TLE provides reliable information on the IPL core lipid distribution (CLs from IPLs), which was composed of *ca*. 80% of diethers and 20% of tetraethers (Figure 3 and Table 3). To evaluate the lipid content per cell based on this CL composition, i.e., lipids without polar head group, an estimation of the nature and relative proportions of *T. barophilus* polar head groups is, however, required. The dedicated analysis of the polar head groups only allowed to access a few, low mass hexose derivatives (*ca.* 242 Da with a phosphatidyl group). Although none was detected here, a larger range of polar head groups, including smaller, e.g., PE (123 Da), and substantially larger ones, e.g., PHexHex2NAc (486 Da), sulfonotri- and tetrahexoses (551 and 713 Da), is to be expected in archaea (Kates, 1993; Jensen et al., 2015). This suggests that the polar head group can represent a non-negligible mass proportion of a given IPL, which we hypothesized to range from 0.2 (e.g., only small head groups like one and two PE for diether and tetraethers lipids, respectively) to 1 (e.g., only massive head groups like one and two phosphatidyltetrahexose for diether and tetraethers lipids, respectively) times the mass of the core lipid. Considering putative low (1:0.2) and high (1:1) mass ratios between the core lipid and the polar head group and the quantity of core lipids retrieved upon acid methanolysis (Table 3), we estimated that our TLE actually contained from 0.1 to 0.2 fg of lipids per cells. The 18 IPLs identified with UHPLC-MS (0.12 fg cell^–1^) thus represented 50–100% of the TLE (Figure 4).

**TABLE 3:** Core lipid composition of the biomass (totCLs) and of the total lipid extract (CLs from IPLs) of *T. barophilus*.

	Diethers^a^						Tetraethers^a^						Total	
	DGD I			PSGD XII			GDGT0 XVIII			GTGT0 XXII				
					
	μ g	fg cell^–1^	Rel%	μ g	fg cell^–1^	Rel%	μ g	fg cell^–1^	Rel%	μ g	fg cell^–1^	Rel%	fg cell^–1^	D/T
TotCLs	22.2	0.1	28	6.1 × 10^–2^	3.1 × 10^–4^	Traces	56.8	0.3	72	0.4	2.2 × 10^–3^	Traces	0.4	0.39
CLs from IPLs	14.6	7.3 × 10^–2^	80	4.2 × 10^–2^	2.1 × 10^–3^	Traces	3.6	1.8 × 10^–2^	20	3.1 × 10^–2^	1.5 × 10^–4^	Traces	9.1 × 10^–2^	4.0

*DGD, dialkyl glycerol diethers; PSGD, phytanylsesterterpanyl glycerol diethers; GDGT0, glycerol dialkyl glycerol tetraethers with no cyclopentane ring; GTGT0, glycerol trialkyl glycerol tetraethers with no cyclopentane ring; D/T, diethers over tetraethers ratio; Rel%, molar relative proportion; μg, absolute lipid quantity in μg; fg cell^–1^, cellular lipid abundance in fg cell^–1^ calculated with an average cell number of 2.0 × 10^8^ cell mL^–1^; ND, not detected. Traces, < 1%. ^a^Relative proportions account for protonated adducts in APCI positive mode and were calculated assuming a response factor of 0.42 for diethers and 0.57 for tetraethers relative to the internal standard C_46_-GTGT, or of 0.74 for diethers relative to tetraethers (refer to methods).*

To further estimate how close this lipid composition was from the real lipidome of *T. barophilus*, we compared it with calculated and an experimentally derived theoretical total lipid contents (refer to Figure 4 for a summary of these estimations). *T. barophilus* cells are coccoid with a cell diameter of 0.8 to 2.0 μm (Marteinsson et al., 1999) and covered in one or more dense proteinaceous surface layers like other Thermococcales (Kostyukova et al., 1999). Using the calculations described by Lipp et al. (2008) with a membrane thickness of 5.5 nm and protein content of 70%, our calculated theoretical total lipid content ranged between 3.0 and 20 fg cell^–1^. The direct acid methanolysis of *T. barophilus* biomass yielded its total core lipid content (total CLs), which contained *ca*. 30% of diethers and 70% of tetraethers (Table 3). As for the estimation of the TLE content, considering low and high mass ratios between the core lipid and the polar head group resulted in an experimentally derived theoretical total lipid content of *T. barophilus* ranging from 0.5 to 0.8 fg cell^–1^. Our TLE (0.1 to 0.2 fg cell^–1^) thus represented *ca*. 12–40% of *T. barophilus* lipidome (experimentally derived theoretical lipid content; Figure 4).

### Matrix-Assisted Laser Desorption/Ionization Fourier Transform Ion Cyclotron Resonance Mass Spectrometry Reveals Novel Tetraether-Based Intact Polar Lipids in *Thermococcus barophilus*

**FIGURE 5:**
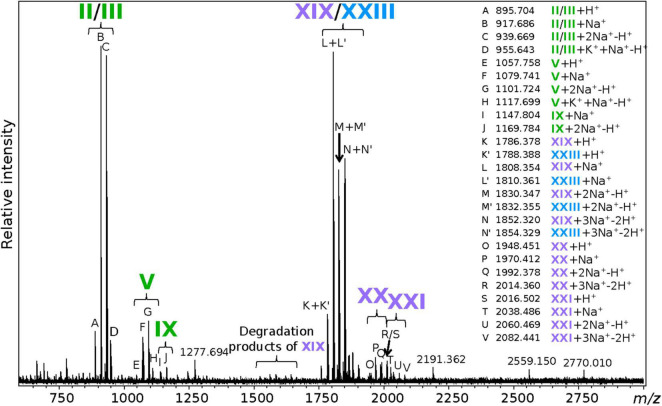
Extraction-free analysis reveals novel tetraether-based intact polar lipids in *Thermococcus barophilus*. m/z detected for each marked peak and the putative corresponding lipid adduct are listed on the right side of the figure. Masses similar to that expected from partial hydrolysis of tetraethers, e.g., PI-GDGT0-P, PI-GDGT0 and P-GDGT0, are indicated as degradation products of PI-GDGT0-PI XIX. The masses of major unidentified peaks are also displayed. Refer to Figure 1 for lipid structures.

Matrix-Assisted Laser Desorption/ionization Fourier Transform Ion Cyclotron Resonance MS (MALDI-FT-ICR-MS) allows for the direct ionization of a sample, without prior wet-chemical treatment, and thus offers a valuable tool to explore archaeal IPLs that are unreachable by the extraction-based analytical procedures. However, it is worth noting that other biomolecules besides lipids can be ionized and detected when analyzing cell pellets directly. Thus, special attention must be paid when attributing a compound to a peak. Here, lipid structures were determined based on the molecular masses (| mass error| ≤ 50 ppm) of the different adducts detected and, when possible, on the comparison with lipids detected in the TLE by UHPLC-MS (Figure 2). An analysis of *T. barophilus* TLE using MALDI-FT-ICR-MS resulted in clusters of peaks (Supplementary Figure 12), suggesting the presence of numerous isotopologs and singly charged adducts, e.g., [M + H]^+^, [M + Na]^+^, [M + K]^+^, [M + 2Na-H]^+^, [M + K + Na-H]^+^, [M + 3Na-2H]^+^, and [M + 2K-H]^+^, the monosodiated adduct being always the most abundant. Only PI-DGD II and/or PGlc-DGD III could be identified from the TLE, which confirmed its high abundance as observed in UHPLC-MS and the possibility to detect *T. barophilus* IPLs using MALDI-FT-ICR-MS. In contrast, a direct ionization of *T. barophilus* biomass under optimized experimental conditions (not shown) revealed the majority of *T. barophilus* most abundant diether-based IPLs identified in UHPLC-MS, i.e., PI-DGD II and/or PGlc-DGD III, PGlcGlc-DGD V, and PGlcHex + C_5_H_8_-DGD IX, although some were not observed, e.g., PGlcNAc-DGD VI. A special focus was paid to specifically target *T. barophilus* unidentified tetraether lipid diversity, and PI-GDGT0-PI XIX and PI-GTGT0-PI XXIII indeed appeared in a seemingly much higher abundance than that revealed by UHPLC-ESI-MS (Figure 5). Other, lower mass ion adducts were detected, e.g., at *m/z* = 1646.304, 1566.339, and 1324.390, and were assigned to the sodiated adducts of PI-GDGT0-P, PI-GDGT0, and GDGT0 XVIII, respectively. As those compounds were not identified in *T. barophilus* TLE analyzed with UHPLC-ESI-MS, they were assumed to result from laser-induced partial degradation of the much more abundant PI-GDGT0-PI XIX (Figure 5). Additionally, two putative uncharacterized tetraether-based IPLs were observed, namely compounds XX and XXI. Compound XX showed molecular ions at *m/z* = 1948.451, 1970.413, and 1992.396 that could be assigned to the [M + H]^+^, [M + Na]^+^, and [M + 2Na-H]^+^ adducts of PHex-GDGT0-PHexHex (theoretical ions at *m/z* = 1948.414, 1970.396, and 1992.378, respectively). Similarly, molecular ions of compound XXI at *m/z* = 2016.483, 2038.485, and 2060.468 were assigned to [M + H]^+^, [M + Na]^+^, and [M + 2Na-H]^+^ adducts of PHex-GDGT0-PHexHex + C_5_H_8_ (theoretical ions at *m/z* = 2016.476, 2038.458, and 2060.440, respectively). Despite a relatively large gap between theoretical and observed masses (mass error = -20.7 ± 11.8 ppm) which might result from very low amounts of those compounds and the increasing absolute mass error with higher masses, these putative structures were further supported by the detection of 2Hex polar head groups in fraction F7 corresponding to the tetraether unresolved peak in UHPLC-MS (Table 2). Other tetraether-based IPLs bearing polar head groups similar to those detected on the diether-based IPLs, such as (poly)N-acetylated hexosamine, were also screened for but not detected. Scanning *T. barophilus* biomass for higher masses neither yielded other ions nor improved the recovery of the newly identified IPLs (not shown).

Altogether, 18 saturated and 64 unsaturated IPLs were detected and tentatively identified in *T. barophilus*. The structures of PI-DGD II, PGlc-DGD III, PGlcNAc-DGD IV, PGlcGlc-DGD V, PGlcHexNH_2_-DGD VI, PGlcHex + C_5_H_8_-DGD IX, PI-PSGD XIII, PGlcGlc-PSGD XIV, PGlcHexNH_2_-PSGD XV, and PI-GDGT0-PI XIX were validated by analyzing independently their polar head groups and core structures, whereas those of PHexHexNAc-DGD VII, PHexHex2NAc-DGD VIII, CDP-DGD XI, and PI-MeDGD XVII were determined based on their fragmentation patterns alone. In contrast, the structure of PI-GTGT0-PI XXIII was derived solely from the acid hydrolysis results, whereas those of PHex-GDGT0-PHexHex XX and of PHex-GDGT0-PHexHex + C_5_H_8_ XXI resulted from the molecular masses detected by FT-ICR-MS.

## Discussion

### Novel Intact Polar Lipid Structures Were Uncovered From the Diverse Lipid Composition of *Thermococcus barophilus*

By means of UHPLC-MS and MALDI-FT-ICR-MS, 18 saturated and 64 unsaturated IPLs were identified in *T. barophilus* (Table 1). Fourteen IPLs were based on diethers, i.e., DGD I, PSGD XII, and MeDGD XVI, and four on tetraethers, i.e., GDGT0 XVIII and GTGT0 XXII. Ten distinct polar head groups were detected, among which three were derivatives of phosphatidylhexose (PI, PGlc, PGlcNAc), six of glycosylated phosphatidylhexose (PGlcGlc, PGlcHexNH_2_, PHexHexNAc, PHexHex2NAc, PGlcHex + C_5_H_8_, PHexHexNH_2_ + C_5_H_8_), and one of nucleoside diphosphate (CDP; Table 1).

Similarly to numerous other archaea, and especially Thermococcales (De Rosa et al., 1987; Lanzotti et al., 1989; González et al., 1998; Lobasso et al., 2012; Meador et al., 2014), PI-DGD II was the dominant IPL of *T. barophilus*. Whereas PSGD-based IPLs were reported in numerous halophilic archaea and a few methanogens [see, for instance (Koga et al., 1993a; Hezayen et al., 2001; Becker et al., 2016; Bale et al., 2019)], this study reported for the first time the presence of PSGD and MeDGD-based IPLs in Thermococcales and in hyperthermophilic archaea. In addition to *T. barophilus*, PI-GTGT0-PI XXIII has been reported in *Thermococcus kodakarensis*, *Pyrococcus furiosus*, and *P. yayanosii* (Tourte et al., 2020a) and may be a common IPL to all Thermococcales, as the core lipid GTGT0 XXII was reported in every Thermococcales investigated so far (Tourte et al., 2020b). Glucose has been reported repeatedly as a major sugar residue in archaeal glycolipids [see, for example, (Lanzotti et al., 1987; Ferrante et al., 1990; Trincone et al., 1992; Koga et al., 1998; Cui et al., 2012)], but only rarely in phosphoglycolipids, i.e., in *Aeropyrum pernix* (Sako et al., 1996) and in *Thermococcus zilligi* (Lanzotti et al., 1989), a close relative of *T. barophilus*. Meador et al. (2014) recently updated the IPL composition of *T. kodakarensis* and notably identified PHexNAc-DGD, PHexHex-DGD, PHexHexNH_2_-DGD, and PHexHexNH_2_ + C_5_H_8_-DGD, with no further characterization of the polar head groups. To our knowledge, our detailed investigation of the sugar residues is the first report of such a diversity of glucose derivatives as polar head groups of phosphoglycolipids in archaea, which were initially assumed to be mostly built upon inositol. This study also reported for the first time mono- and diacetylated PHexHex-DGD VII and VIII as well as PHexHex + C_5_H_8_ as a GDGT0 polar head group in XXI. Although our results do not drastically contrast with the lipid composition of other Thermococcales, they extend the known lipid diversity for this order of archaea and beyond and place *T. barophilus* as a prime model for further investigation of the Thermococcales membrane composition, organization, and adaptation. In particular, *T. barophilus*, among other model archaea, was shown to alter its core diether/tetraether ratio with growth temperature and hydrostatic pressure (Cario et al., 2015). The updated IPL compositions reported here for the hyperthermophilic and piezophilic archaeon *T. barophilus* grown under optimal laboratory conditions (85°C and atmospheric pressure) now pave the way for elucidating how archaea adapt their lipidomes under varying conditions. Lipidome changes induced by high hydrostatic pressures would be worth special attention since such membrane adaptations remain so far mostly unexplored.

### A Combination of Ultra-High Performance Liquid Chromatography-Mass Spectrometry and Matrix-Assisted Laser Desorption/Ionization Fourier Transform Ion Cyclotron Resonance-Mass Spectrometry to Elucidate *Thermococcus barophilus* Intact Polar Lipid Composition

The archaeal lipid extraction and fractionation have previously been demonstrated to be biased toward certain lipid classes (Huguet et al., 2010; Cario et al., 2015; Law and Zhang, 2019). The first and only description of *T. barophilus* intact polar lipids, which reported exclusively PI-DGD II (Marteinsson et al., 1999), undoubtedly suffered from such biases. The reevaluation of *T. barophilus* CLs indeed showed an abundance of tetraether-based IPLs and demonstrated the impossibility to exhaustively extract its IPLs with typical extraction procedures (Cario et al., 2015). While the reassessment of *T. barophilus* IPLs in this study did provide a greater insight into its diversity, including tetraether-based IPLs (Table 1), we could only access 0.12 fg of lipid cell^–1^. Although this remains in line with observations made for other archaea, e.g., *T. barophilus* close relative *T. kodakarensis* [0.38 to 1.61 fg cell^–1^ (Meador et al., 2014)], our results highlighted three limitations of the current procedure that can explain such a low yield.

First, we observed a large difference between *T. barophilus* calculated and experimentally derived theoretical lipid contents (3.0 to 20.0 vs. 0.5 to 0.8 fg cell^–1^), which suggests that our calculations might be far off *T. barophilus* real total lipid content. Indeed, even adding up the other lipid molecules, *T. barophilus* synthesize and that are not visible after methanolysis of the biomass, i.e., polyprenyl phosphates (Supplementary Figure 11) and apolar polyisoprenoids [up to 1% of the membrane content (Cario et al., 2015)] to our experimentally derived theoretical total lipid content would not be enough to reach the calculated theoretical total lipid content. Our results obtained with rough estimates of *T. barophilus* cell diameter and membrane lipid/protein ratio combined with those obtained for other archaea such as *M. thermautotrophicus* (Yoshinaga et al., 2015) and *T. kodakarensis* (Meador et al., 2014) thus suggest that the model for cell lipid content, initially built for bacterial cells and congruent with their physiological parameters (Simon and Azam, 1989), requires revisions to better fit the archaeal cell membrane. Although *T. barophilus* experimentally derived theoretical total lipid content might also be inaccurate, for instance due to other unidentified polar head groups larger than the phosphatidylmono- and di-hexoses considered here, it was used hereafter as a reference (Figure 4). While closer to the total IPLs detected, the experimentally derived theoretical total lipid content still showed a large discrepancy with what we could actually access with our extraction method (0.5 to 0.8 vs. 0.12 fg cell^–1^), suggesting that other biases might hinder the elucidation of *T. barophilus* entire lipidome.

We observed a major discrepancy between this experimentally derived theoretical value and extracted lipid contents (0.5 to 0.8 vs. 0.1 to 0.2 fg cell^–1^; Figure 4), which highlights the inefficiency of our extraction procedure to recover *T. barophilus*’ lipids. Two non-exclusive hypotheses could explain the low yield of our extraction method: (1) either *T. barophilus* cells and/or (2) lipids are especially resistant to our extraction procedure. On the one hand, Thermococcales cells, including *T. barophilus*, were shown to harbor at least one quasicrystalline protein surface layer (Kostyukova et al., 1999; Marteinsson et al., 1999), which might prevent proper cell lysis and thus lipid extraction. This is congruent with our microscopic observation of intact-looking cells in the cell debris and our ability to sometimes recover cell growth even after lipid extraction with the Blye and Dyer organic solvents (unpublished observations). In addition, the physiological state (e.g., growth stage and cell morphology) of *T. barophilus* might also enhance this resistance. On the other hand, our TLE contained only *ca*. 20% of tetraethers (Table 3), whereas they represented from 45 to 70% of *T. barophilus* experimentally derived theoretical total lipid content in previous (Cario et al., 2015; Tourte et al., 2020b) and in this study, respectively. Despite inconsistencies in the total amount of tetraethers *T. barophilus* synthesizes that might stem from different growth conditions, physiological states, or extraction/analytical conditions, all three studies agree on a large discrepancy of diether/tetraether distributions between the experimentally derived theoretical total lipid content and the TLE. This suggests that most of the IPLs not recovered are built upon tetraethers, and thus supports a major deficiency of our extraction procedure in recovering tetraether-based IPLs.

We also highlighted a minor inconsistency between extracted and detected IPLs (0.1 to 0.2 vs. 0.12 fg cell^–1^), which suggests that part of the IPLs that are indeed extracted might remain invisible to our detection method. Despite being composed of up to 20% of tetraethers (Table 3), *T. barophilus* TLE displayed only two tetraether-based IPLs upon UHPLC-ESI-MS analysis, i.e., PI-GDGT0-PI XIX and PI-GTGT0-PI XXIII, which represented less than 1% of the TLE (Table 2). Although a fraction of tetraether-based IPLs are indeed extracted and present in the TLE, these results suggest that they remain resistant to detection by our analytical setup. Additionally, *T. barophilus* TLE was overwhelmingly dominated by PI-bearing IPLs both in this study (90%, Figure 4 and Table 1) and that of Marteinsson et al. (1999) (100%), while neither glycolipids nor other polar head groups typically found in archaea [e.g., PE, PS and PG, (Koga et al., 1987; Trincone et al., 1992; Kamekura and Kates, 1999; Meador et al., 2014; Jensen et al., 2015)] were observed. It is now widely accepted that physicochemical properties and physiological and adaptive functions of biological membranes are governed by the structural diversity of both the alkyl chains and the polar head groups found in the lipidome (Kučerka et al., 2015). One may thus speculate that a natural membrane containing almost exclusively one polar head group might not be biologically functional. While the absence of typical archaeal IPLs in *T. barophilus* might be linked to its particular membrane physiology and/or environmental conditions, a functional membrane composed of >90% of a single IPL is hardly conceivable, and *T. barophilus* membrane should theoretically contain other isomers and derivatives of phosphatidyl(poly)hexoses not detected here to be functional. This therefore suggests that our UHPLC-ESI-MS analytical procedure, and probably our extraction method as well, might artificially enhance the detection of PI based over other phosphatidylhexose-derivative IPL populations. Altogether, our results thus highlight two major shortcomings of our extraction and analytical procedure, i.e., preferential extraction and detection of (1) diether-based and (2) PI-bearing IPLs, which resulted in a *T. barophilus* lipidome artificially composed of almost exclusively PI-DGD II. However, our study shows that there is still much to explore in the lipidome of *T. barophilus* and provides clues about the presence of other lipids such as tetraethers with derivatives of phosphatidyl(poly)hexoses probably based on distinct sugar moieties.

In contrast to UHPLC-MS, MALDI-FT-ICR-MS of *T. barophilus* cell pellet revealed high levels of several tetraether lipids when focusing on high *m*/*z*. For instance, PI-GDGT0-PI XIX appeared as one of *T. barophilus* main IPLs with our MALDI-FT-ICR-MS settings (Figure 5). In addition to PI-GDGT0-PI XIX, other low-mass tetraether derivatives such as PI-GDGT0-P, PI-GDGT0, and P-GDGT0 were identified (Figure 5). PI-GDGT0 has been repeatedly reported in archaea, including Thermococcales (Sprott et al., 1997; Meador et al., 2014; Tourte et al., 2020a), but almost always using rather destructive ionization methods [for instance, fast-atom bombardment in Sprott et al. (1997). Similarly, PI-GDGT0 was only detected here with the MALDI-FT-ICR-MS under the highest laser power setting (not shown) and not with our soft UHPLC-ESI-MS method (Figure 2). This suggests that our MALDI-FT-ICR-MS procedure might alter tetraether lipids, preventing their detection as IPLs, and that the aforementioned compounds could stem from laser-induced degradation rather than be true IPLs of *T. barophilus*. MALDI-FT-ICR-MS nonetheless allowed to access previously unknown tetraether-based IPLs, i.e., PHex-GDGT0-PHexHex XX and PHex-GDGT0-PHexHex + C_5_H_8_ XXI. Altogether, these results proved MALDI-FT-ICR-MS to be a prime alternative to UHPLC-MS for exploring archaeal lipid diversity and especially tetraether-based IPLs. In the future of MALDI-FT-ICR-MS lipidomics, fine tuning of the laser parameters, including laser-based post-ionization (Ellis et al., 2018), and of the matrix composition should help access an even wider archaeal IPL diversity, although the combination with UHPLC-MS remains necessary for lipid quantitation and to elucidate the complete lipidome of archaea.

### Insights Into *Thermococcus barophilus* Membrane Organization

Most of the physical properties of archaeal lipids were based on the study of synthetic PE- and PC-bearing lipids, and the absence of a comprehensive inventory of *T. barophilus*’ polar head groups prevented further understanding of its membrane physicochemical properties and organization. The detection of 82 IPLs, however, opens new avenues for understanding the membrane contribution and biological functions of archaeal polar head groups as even the least abundant lipids were shown to ensure key cellular functions in both bacteria and archaea (Mileykovskaya and Dowhan, 2009; Kellermann et al., 2016).

PI, present in PI-DGD II, PI-PSGD XIII, PI-MeDGD XVII, PI-GDGT0-PI XIX, and PI-GTGT0-PI XXII, was the most abundant polar head group in *T. barophilus* TLE (Table 1). Physical studies using neutron and x-ray diffractions and NMR spectrometry demonstrated that PI extended deeply into the aqueous molecular environment in a slightly tilted configuration relative to the membrane surface normal (McDaniel and McIntosh, 1989; Hansbro et al., 1992; Bradshaw et al., 1999). The extension of PI away from the membrane surface favors intra- and intermolecular hydrogen bonds, thus creating an extensive network that shields the membrane with a large electrostatic barrier preventing proton and ion leakages (Hinz et al., 1985; McDaniel and McIntosh, 1989; Bradshaw et al., 1999). In addition, the direct projection of PI into the aqueous molecular environment allows for a maximum hydration of the inositol ring (Bradshaw et al., 1999), which turns into a bulky hydrated head group. The high volume of this bulky head group relative to that of the lipid alkyl chains enhances the conformational freedom of the latter, which might eventually result into a looser packing and a higher water permeation in model membranes containing PI (Peng et al., 2012). This packing defect generated by the PI head group, especially in the tightly packed archaeal isoprenoid membranes, would in turn unlock more loading space for membrane proteins and the higher water permeation would allow for solvent interactions essential to protein stability within the membrane environment (Peng et al., 2010, 2012). In contrast, the bulky head group creates a repulsive hydrated layer that stabilizes the membrane by preventing deformation and membrane fusion (Sundler and Papahadjopoulos, 1981; Kanduč et al., 2017). The various membrane macrostructures observed in Thermococcales, e.g., nanotubes and vesicles (Marguet et al., 2013; Gorlas et al., 2015), should theoretically be greatly disfavored if their membrane was indeed composed exclusively of PI-based lipids that prevent membrane remodeling. The data further confirm our assumption that PI might not necessarily be the major head group in *T. barophilus* despite its overwhelming dominance in our extracts (Table 1). In contrast, low proportions of PI would provide an enhanced fluidity in an otherwise tightly packed archaeal membrane while preserving its impermeability.

In addition to inositol, glucose was found in a variety of polar head group derivatives that represented *ca.* 8% of *T. barophilus* TLE (Table 1). Due to the structural similarities between hexose isomers, one might speculate that their effects on biological membranes would be comparable but synthetic glycolipids bearing distinct hexose moieties showed different orientations relative to the membrane surface. Glc nonetheless displayed an extended conformation similar to that of Ino (McDaniel, 1988), suggesting that it might support membrane physicochemical properties analogous to those described above.

Stereochemical changes of a single hydroxyl group were shown to dramatically alter membrane properties and stability (Hinz et al., 1991; Róg et al., 2007). Various derivatives of glucose bearing distinct additional groups were identified in *T. barophilus* (Table 1), but their exact position on the hexose ring and the presence of different position isomers could not be ascertained although they might support distinct functions. For instance, positions of the phosphatidyl groups in phosphoinositides impacted their ionization properties, hydrogen bond networks, and thus their interactions with membrane proteins and lipids (Kooijman et al., 2009). No data are currently available on the alterations of the lipid properties generated by the additional NAc, NH_2_, and C_5_H_8_ moieties detected in *T. barophilus*. However, an O-acetylation on the C-6 atom of the glucose ring of a fatty-acyl analog of PGlc-DGD III found in various bacterial and mammalian cells was demonstrated to change the immunogenic properties of the IPL, e.g., a eukaryotic anti-phosphatidylglycoside antigen reacted more strongly to phosphatidyl glucopyranoside than to its O-acetylated homolog (Nagatsuka et al., 2006; Takahashi et al., 2012), suggesting that NAc-bearing IPLs of *T. barophilus* might exert different properties than their hydroxylated forms. Based on the polarity of these moieties, one might also speculate that NAc and NH_2_ would behave similarly than the regular hydroxyl group, whereas the apolar C_5_H_8_ could cause dramatic changes in the orientation, hydration, and interaction of the monosaccharide ring.

The polar moiety of lipids bearing diglycosides has been shown to extend away from the surface and to generate intermolecular hydrogen bonds, hence conferring the diglycosidic lipids similar physicochemical properties than those of monoglycosidic ones (Jarrell et al., 1987; Renou et al., 1989; Polak et al., 2014). In addition, the even higher relative volume of the polar head group might result in further enhanced conformational freedom of the alkyl chain. Derivatives of glycosylated phosphatidylhexose lipids, such as PHexHex, PHexHexNH_2_, and PHexHex + C_5_H_8_, have been detected in bacteria, eukarya, and archaea (Nishihara et al., 1992; Meador et al., 2014). Although their biological purpose remains elusive in archaea, these lipid derivatives notably act as protein anchor in bacterial and eukaryotic membranes (Low and Saltiel, 1988), and similar functions might be expected for their archaeal counterparts.

Altogether, these results enhance our comprehension of the membrane organization suggested for *T. barophilus* and highlight putative biological functions for the different IPLs detected in this study. Characterization of the physicochemical properties of synthetic or natural archaeal lipids with phosphoglycosidic head groups remains nonetheless essential to precisely define the role of the diverse archaeal lipid compositions in membrane physiology and organization.

## Conclusion

We reassessed here the intact polar lipid, core lipid, and lipid polar head group compositions of *Thermococcus barophilus*, a model for membrane architecture and adaptation to extreme conditions in archaea. We unraveled the presence of at least 82 distinct membrane lipids, including a variety of core structures, i.e., saturated and unsaturated DGD, MeDGD, PSGD, GDGT, and GTGT, and the widest diversity of polar head groups in Thermococcales known to date. Although not drastically different from that of *T. barophilus* close relatives, the lipid composition reported here extends the known diversity of phosphoglycosidic head groups known in Thermococcales. In agreement with previous investigations of *T. barophilus* and other Thermococcales IPLs, the lipid diversity revealed here was overwhelmingly dominated by PI-DGD. The low extraction yield, the excessive prevalence of PI-DGD, the low diversity of polar head group moieties compared to bacterial and eukaryotic lipidomes (although high compared to other archaeal lipidomes), and the CL released upon acid methanolysis demonstrated that a large portion of *T. barophilus* lipidome still remains inaccessible to the employed extraction and analytical protocols. Extraction-free analysis with MALDI-FT-ICR-MS allowed to access previously undetected tetraether-based IPLs, and further improvements and developments of new methodologies might pave the way to the discovery of completely new archaeal IPLs. Due to the isoprenoid alkyl chains of archaeal lipids, *T. barophilus* membrane is tightly packed, but the addition of bulky phosphatidylhexose head groups might provide relaxation while maintaining impermeability. Altogether, our results illustrate the complexity and diversity of *T. barophilus* membrane structure and pose this species as a prime model to elucidate archaeal membrane lipid diversity, properties, and organization.

## Data Availability Statement

The original contributions presented in this study are included in the article/Supplementary Material, further inquiries can be directed to the corresponding author.

## Author Contributions

K-UH and PO: conceptualization, funding acquisition, project administration, and supervision. MT and SC: formal analysis. MT, SC, and LW: investigation. MT, SC, JL, and LW: methodology. MT: visualization and writing – original draft. MT, SC, LW, JL, K-UH, and PO: writing – review and editing. All authors contributed to the article and approved the submitted version.

## Conflict of Interest

The authors declare that the research was conducted in the absence of any commercial or financial relationships that could be construed as a potential conflict of interest.

## Publisher’s Note

All claims expressed in this article are solely those of the authors and do not necessarily represent those of their affiliated organizations, or those of the publisher, the editors and the reviewers. Any product that may be evaluated in this article, or claim that may be made by its manufacturer, is not guaranteed or endorsed by the publisher.

## Abbreviations

IPL: intact polar lipid(s)
CL: core lipid(s)
MGD: monoalkyl glycerol diethers
DGD: dialkyl glycerol diethers
PSGD: phytanylsesterterpanyl glycerol diethers
MeDGD: methylated DGD
GDGT: glycerol dialkyl glycerol tetraethers
GTGT: glycerol trialkyl glycerol tetraethers
MGDG: monogalactosyl diacylglycerol
DGDG: digalactosyl diacylglycerol
C_46_-GTGT: GTGT containing 46 carbon atoms
PI: phosphatidyl inositol
PHex: phosphatidyl hexose
PHexNAc: phosphatidyl N-acetylhexosamine
PHexHex: glycosylated phosphatidyl hexose
PHexHexNH_2_: hexosamine phosphatidyl hexose
PHexHexNAc: N-acetylhexosamine phosphatidyl hexose
PHexHex2NAc: di-N-acetylhexosamine phosphatidyl hexose
PHexHex + C5H8: glycosylated phosphatidyl hexose bearing an additional mass of 68
CDP: cytidine diphosphate
PG: phosphatidyl glycerol
C_21_-PC: phosphatidyl choline diacylglycerol with two C_21_ fatty acid chains
MeOH: methanol
DCM: dichloromethane
ACN: acetonitrile
TFA: trifluoroacetic acid
FA: formic acid
NH_3(aq)_: ammonium hydroxide or aqueous ammonia
DHB: 2,3-dihydroxybenzoic acid
UHPLC: ultra-high performance liquid chromatography
Q-TOF: quadrupole time of flight
QQQ: triple quadrupole
MS: mass spectrometry
ESI: electrospray ionization
APCI: atmospheric-pressure chemical ionization
MALDI: matrix-assisted laser desorption/ionization
FT: Fourier transform
ICR: ion cyclotron resonance.
